# ALK-negative lung inflammatory myofibroblastic tumor in a young adult: A case report and literature review of molecular alterations

**DOI:** 10.1097/MD.0000000000025972

**Published:** 2021-05-21

**Authors:** Silvia Angela Debonis, Alberto Bongiovanni, Federica Pieri, Valentina Fausti, Alessandro De Vita, Nada Riva, Lorena Gurrieri, Silvia Vanni, Danila Diano, Laura Mercatali, Toni Ibrahim

**Affiliations:** a Osteoncology and Rare Tumors Center, IRCCS Istituto Romagnolo per lo Studio dei Tumori (IRST) “Dino Amadori,” Meldola; b Pathology Unit, “Morgagni-Pierantoni” Hospital, Forlì; c Radiology Unit, IRCCS Istituto Romagnolo per lo Studio dei Tumori (IRST) “Dino Amadori,” Meldola, Italy.

**Keywords:** anaplastic lymphoma kinase, case report, inflammatory myofibroblastic tumor, young adult

## Abstract

**Rationale:**

Inflammatory myofibroblastic tumor (IMT) is a rare mesenchymal tumor that is prevalent among children and adolescents. Surgery is the most important therapeutic approach for IMT and complete resection is recommended. Although 50% of IMTs show anaplastic lymphoma kinase (ALK) rearrangements, crizotinib has proven an effective therapeutic approach. However, the genetic landscape of this tumor is still not fully understood and treatment options are limited, especially in the majority of ALK-negative tumors.

**Patient concerns:**

We describe the clinical case of a healthy 18-year-old female in whom a pulmonary nodule was incidentally detected

**Diagnoses:**

Following a small increase in the size of the nodule, the patient underwent both ^18^FDG-PET/CT and ^68^Ga-PET/CT, resulting in a suspicion of bronchial hamartoma.

**Interventions:**

The patient underwent surgery and a salivary gland-like lung tumor was diagnosed.

**Outcomes:**

After surgery, the patient was referred to our cancer center, where a review of the histology slides gave a final diagnosis of ALK-negative lung IMT. Given the histology, it was decided not to administer adjuvant therapy and the patient was placed in a 3-monthly follow-up program. The patient is still disease-free 2 years post-surgery.

**Lessons:**

Although there is no standard of care for the treatment of IMT, identifying genomic alterations could help to redefine the management of patients with negative-ALK disease. Our review of the literature on IMT and other kinase fusions revealed, in addition to ALK rearrangements, the potential association of ROS1, NTRK, RET, or PDGFR beta alterations with the tumor.

## 1 Introduction

Inflammatory myofibroblastic tumors (IMTs) are rare mesenchymal tumors with an incidence of 0.04%. IMTs of soft tissue origin are more frequent in females than in males, while lung IMTs show a 1:1 ratio and are usually found in children, adolescents, and young adults, although they can occur at any age.^[1]^

The lungs are the most common site of IMT onset, although the disease can also originate in other anatomic sites such as the retroperitoneum, abdomen, and pelvic cavity. Despite the low metastatic potential, surgery remains the gold standard of care for localized resectable disease. IMTs can also be locally invasive and are associated with distant metastasis in around 10% of cases.^[2,3]^ Overall, however, IMT prognosis is good, with a 5-year survival rate of 74% to 91%.^[4]^

Around 50% of IMT cases harbor a clonal translocation that activates the anaplastic lymphoma kinase (ALK)-receptor tyrosine kinase gene located at 2p23 locus.^[5]^ As a result, ALK protein is overexpressed and can be detected by immunohistochemical tests. ALK acquires an oncogenic potential following gene fusion, as in anaplastic large cell lymphoma, lung cancer and IMT, or as a result of a missense mutation, as in neuroblastoma and anaplastic thyroid cancer.^[6]^

ALK positivity in immunohistochemical testing, which represents *ALK*-based gene fusions, is more prevalent in pediatric IMT patients than in adult cases. However, it is unclear whether this discrepancy is dependent on an intrinsic difference in the biology of IMT between the 2 age groups (child vs adult), or whether it is merely a reflection of a wider spectrum of genetic alterations.^[7,3]^

IMTs display a wide morphologic spectrum ranging from an inflammatory “pseudotumor” with predominant hyalinization, chronic inflammation and few lesional spindle cells, to a highly cellular myofibroblastic proliferation occasionally defined as sarcomatous neoplasm due to the lack of a significant inflammatory or/and fibromyxoid stromal component. The diagnosis of ALK-negative IMTs is often difficult because of its markedly variable phenotype and lack of objective immunoprofile in relation to a potential waste-basket of different entities, including reactive/inflammatory processes such as the fibro-inflammatory IgG4-related disease, idiopathic retroperitoneal fibrosis, and at the other end of the spectrum, other spindle cell/pleomorphic sarcoma.^[8,9]^

Patients with metastatic IMT harboring ALK alterations can be successfully treated with ALK inhibitors.^[10,11]^ However, treatment options are limited for patients with unresectable/advanced disease and for those with ALK-negative tumors.^[12]^

We describe the case of an AYA (adolescent and young adult) patient with ALK-negative lung IMT who underwent surgical resection. We also present our review of the literature on emerging genetic alterations in ALK-negative IMT such as ROS1, NTRK, RET, or PDGFR beta which, as targetable biomarkers, could potentially help to improve the management of patients with ALK-negative disease.

## 2 Case presentation

**Figure 1:**
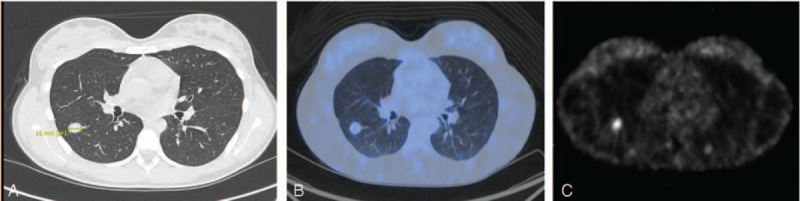
(A) The broncopulmonary nodule detected (diameter 16 mm) was located at the apical segment of of the right lower lobe (RLL). The nodule showed (B) a SUVmax of 3.5 at ^18^FDG-PET/CT and (C) a SUVmax of 3.4 at ^68^Ga-PET/CT. SUV = standardized uptake value.

In December 2015, an 18-year-old girl was admitted to hospital following a car accident. A CT scan performed to exclude the possibility of lung injury incidentally detected a bronchopulmonary nodule with a diameter of 14 mm in the apical segment of the right lower lobe (RLL). The patient was otherwise in good health and specifically denied fever or chills. Her past medical history was unremarkable for inherited diseases. Given the radiological characteristics of the nodule and the young age of the patient, prudential follow up-was opted for in the form of an annual chest CT scan. In December 2018, a slight increase was noted in the longest diameter of the bronchopulmonary nodule (16 × 12 mm compared to the previous 14 × 10 mm). ^18^F-fluoro-2-deoxy-D-glucose- (^18^FDG-) and ^68^Gallium (^68^Ga-) DOTATATE PET/CT scans were performed, revealing an increase in the maximum standardized uptake value of the nodule of 3.5 and 3.4, respectively (Fig. 1).

Endobronchial hamartoma was suspected and, in April 2019, the patient thus underwent videothoracoscopic segmentectomy of the apical segment of RLL with hilar-mediastinal lymphadenectomy. Histopathological examination revealed a complex lesion: the epithelial component showed a well-defined, solid, and acinar architecture without a capsule, with a low degree of malignancy. IHC was positive for CAM5.2, naspin and TTF1, weakly positive for synaptophysin, and negative for chromogranin A, CD56, DOG1 and SOX10. Intense cytoplasmic positivity for PAS was also observed. Eight lymph nodes were negative for metastasis. There was no pleural infiltration. The clinical-morphological and immunophenotypic features were strongly suggestive of salivary gland-like lung cancer.

**Figure 2:**
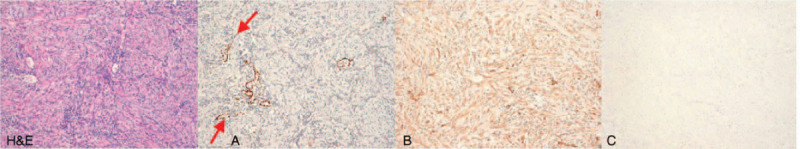
Figures with 10 × magnification show cubic cells **(**hematoxylin and eosin staining H&E) without atypia positive for TTF1 (A) (red arrow), CK7, and napsin and negative for CD117, chromogranin and synaptophysin. IgG plasma cells are numerous, mainly in peripheral areas, but the IgG4 ratio is normal. The spindle cell component is (B) positive for smooth actin and (C) negative for ALK.

After surgery, the patient was referred to our cancer cancer for a second opinion and a revision was made of the histopathology diagnosis by another expert pathologist, as per National Rare Tumor Guidelines. The referral described a central area of compact fascicular spindle cells, sometimes with eosinophilic cytoplasm, mixed with numerous inflammatory elements including plasma cells and lymphocytes inserted in dense fibrous stroma. At the edge of the nodule there were numerous epithelial elements, among which cubic cells without atypia or mitosis, which were immunohistochemically-positive for CK7 and napsin and TTF1 (A) and negative for CD117, chromogranin and synaptophysin. Previously considered as neoplastic, these were probably cells that were reactive to the real lesion. There were numerous IgG plasma cells, mainly in peripheral areas, but the IgG4 ratio was normal. The spindle cell component was positive for smooth actin and negative for ALK using Vysis LSI ALK (2p23) Dual Color, Break Apart Rearrangement probe. Ki67 was 5%. These findings were highly suggestive of an IMT (Fig. 2).The Soft Tissue Sarcoma Multidisciplinary Board of our institute discussed the case, opting for no adjuvant treatment was decided, and the patient was placed in a 3-monthly follow-up program. The patient has been in clinical follow-up for 24 months, with no signs of recurrent disease (Figure S1, Supplemental Digital Content).

## 3 Discussion

IMTs are rare tumors that belong to a subtype of soft tissue sarcoma, with an overall prevalence of 0.04%–0.7%.^[12,13]^ They can occur at any age but are more common in children and adolescents, constituting <1% of adult lung tumors.^[14,15]^

Therapeutic options for patients with unresectable and/or advanced IMT are limited, especially for ALK-negative disease.^[12]^ In addition to surgery, other potentially feasible treatments include radiation and chemotherapy.^[16]^ Dishop et al reported a case of IMT treated with first-line vincristine and etoposide, and subsequently with second-line cisplatin, adriamycin, and methotrexate after incomplete resection.^[17]^ Conversely, Cerfolio et al described 2 cases of IMT in which the residual tumor showed no growth after incomplete resection for the duration of the follow-up (4 and 9 years), neither patient receiving any additional treatment. However, the biological features of the tumors were not reported.^[4]^ Another study reported complete remission in a pediatric IMT patient using vincristine, ifosfamide, doxorubicin, and celecoxib.^[18]^ Steroid and nonsteroidal anti-inflammatory drugs have also been reported as effective for IMT, but the phenotype most likely to benefit is open to debate. In particular, steroids have proven useful for IMT patients with or without IgG4SD features.^[19,8]^ ALK expression and ALK gene rearrangement have been described as good prognostic markers for IMT, whereas ALK-negative IMT appears to be more aggressive, with a higher frequency of metastasis than ALK-positive tumors.^[3,7]^

Among ALK rearrangements in IMT >10 different ALK fusion partner genes have been identified (in IMT), including TPM3/4, RANBP2, TFG, CARS, ATIC LMNA, PRKAR1a, CLTC, FN1, SEC 31A, and EML4. Chromosomal translocation leads to the formation of an ALK fusion protein, which exhibits kinase activity independent of the ligand due to the self-phosphorylation of the chimeric protein. This results in prolonged survival of the cancer cell, hyperproliferation and enhanced cell migration.^[1,20–24]^ In contrast, actionable genomic alterations are not found in around 50% of IMT samples that are immunohistochemically negative for ALK. ALK-negative IMTs may be more aggressive than ALK-positive tumors, with a higher frequency of metastasis.^[3]^

We described a case of slowly-growing ALK-negative IMT in an AYA patient. Initially, there was a misdiagnosis and the patient underwent radiological follow-up for 4 years, with no occurrence of morphological modifications. After surgery, the pathology report was suggestive of a salivary gland-like tumor of the lung, but the final diagnosis of IMT was only made after a review of the original histology slides. This reflects the challenges faced by pathologists in correctly diagnosing ALK-negative IMT.

As little is known about the potential oncogenic drivers of ALT-negative tumors, there are still no targeted therapies available. Recent studies have shown that ALK-negative IMT may harbor other kinase fusions such as ROS1, NTRK, RET or PDGFR beta, giving rise to genome-level research into potential tumorigenic drivers (of this IMT subset). In 2014, a study was published in which other potential IMT actionable targets, including ROS1 and PDGFR beta fusions, were reported for the first time.^[21]^ Genetic analysis was performed by next generation sequencing (NGS) on 33 IMT samples, 11 of which were ALK-negative. In the event of insufficient tumor material, kinase fusions were verified by RNA sequencing. Kinase fusions other than those of ALK were identified in 6 of the 11 ALK-negative samples. Four contained distinct ROS1 fusions (samples L3/L4, YWHAE-ROS1, sample L6, TGF-ROS1), and 2 contained a PDGFR beta fusion (samples L7/L10, NAB2-PDGFR beta). These are both actionable targets of FDA-approved drugs. Of note, ALK fusions were also detected in 2 of the 11 IMT samples that were immunohistochemically negative for ALK expression.^[21]^

ROS1 rearrangements have been reported in around 9% to 13% of IMT, all ALK-negative cases.^[22,25]^ Clinical cases of children/adolescents with pulmonary IMT with a TGF-ROS 1 rearrangement have also been described in the literature, all benefitting from treatment with crizotinib (250 mg) in terms of a significant reduction in tumor size.^[25–27]^

He et al reported, for the first time, a double amplification of CDK4 and MDM2 with protein overexpression by NGS and IHC in a 68-year-old woman with a gastric IMT with local invasion of spleen and diaphragm.^[28]^ Furthermore, Antonescu et al found a correlation between genotype and specific clinical-pathological characteristics of IMT.^[29]^ The authors tested all cases in the study for ALK gene rearrangements ALK-negative tumors were further studied by fluorescence in situ hybridization (FISH) and RNA sequencing for abnormalities in ROS1, PDGFRB, NTRK1, and RET. With regard to the 6 ROS1 rearranged IMTs identified, all except 1 was present in children, located mainly in the lung and intra-abdominal area, and with a specific growth of spindle cells with long cell processes. RET rearrangement was found in a 27-year-old patient with pulmonary IMT characterized by a solid pseudosarcomatous growth and a fatal clinical outcome. Table 1 summarizes the main characteristics of ALK-negative IMT cases reported in the literature.

**Table 1:** Summary of the main case studies on ALK-negative IMT.

Study	Lovly et al (2014)	Mai et al (2019)	Hornick et al (2015)	He et al (2018)	Antonescu et al (2015)
Type of study	Case report N = 1	Case report N = 1	Molecular study N = 30 (9 ALK)	Case report N = 1	Molecular study N = 67 (27 ALK)
Mutations	TGF-ROS1	TGF-ROS1	ROS1 (TGF-ROS1 fusion, YWHAE-ROS1 fusion, fusion partner unknown)	Double amplification of CDK4 and MDM2	ROS1, RET
Treatment	Crizotinib (250 mg)	Crizotinib (250 mg)	no treatment	no treatment	no treatment
Results	Continued decrease in tumor burden	Continuous remission with significant reduction in tumor size	not applicable	not applicable	not applicable
Follow-up	16, 4 mo	5 mo^∗^	NR	8 mo	NR

As previously mentioned, IMT has a higher incidence in children, adolescents and young adults. Our case was an asymptomatic 18-year-old female. Initially, the radiological characteristics of the nodule, the lack of risk factors for neoplastic disease and the young age induced clinicians to monitor the lung nodule radiologically, and it was decided to perform surgery only after an increase in the size of the nodule was observed. Our report highlights the fact that a different diagnostic and therapeutic approach is often needed between AYA and adult patients, indicating the need for more specialized AYA centers in a cancer setting.

## 4 Conclusions

We reported a rare case of ALK-negative lung IMT that was surgically resected. No other gene rearrangements have been investigated to date other than that for ALK. However, from the literature data available, it would appear that the majority of IMTs show kinase fusions. Conventional detection methods such as IHC and FISH may fail to detect a range of actionable gene rearrangements including ROS1, RET, NTRK, and PDGFRβ in IMTs, indicating the need for broader molecular testing, for example, NGS, to explore genetic characteristics more fully. Such an approach could represent an important clinical resource in terms of increasing the number of potential drug targets for patients with IMT, especially those with ALK-negative tumors.

## Acknowledgments

The authors thank Gráinne Tierney and Cristiano Verna for editorial assistance.

## Author contributions

**Data curation:** Federica Pieri, Valentina Fausti, Alessandro De Vita, Nada Riva, Lorena Gurrieri, Silvia Vanni, Danila Diano, Laura Mercatali.

**Supervision:** Alberto Bongiovanni, Toni Ibrahim.

**Validation:** Toni Ibrahim.

**Writing – original draft:** Silvia Angela Debonis, Alberto Bongiovanni.

## Supplementary Material

Supplemental Digital Content
