# Supplementary figures and images for: ALK-negative lung inflammatory myofibroblastic tumor in a young adult: A case report and literature review of molecular alterations
Source: Medicine (Baltimore). 2021 May 21;100(20):e25972. doi: 10.1097/MD.0000000000025972 (PMC8137108; doi:10.1097/MD.0000000000025972)

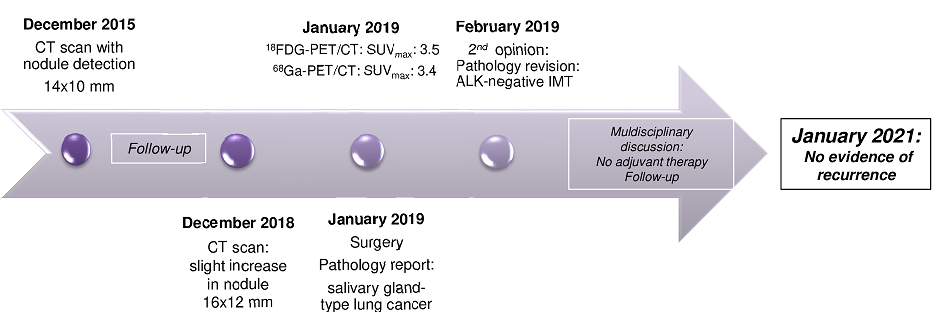

Supplement: Supplemental Digital Content [file medi-100-e25972-s001.doc]
